# A Practical Treatise on Inflammation of the Uterus and Its Appendages, and on Ulceration and Induration of the Neck of the Uterus

**Published:** 1850-06

**Authors:** 


### BIBLIOGRAPHICAL NOTICES.

---

*A Practical Treatise on Inflammation of the Uterus and its appendages, and on ulceration and induration of the Neck of the Uterus.* By James Henry Bennett, M. D., Member of the Royal College of Physicians; Physician Accoucheur to the Western General Dispensary, &c. &c. Second American, from the second London edition. Philadelphia. Lea & Blanchard, 1850.

We have here presented to us a reprint of a volume already well known and well received by the profession on its previous issue; and though nominally a second edition, it is in reality a new work. In it is presented to the reader a complete history of inflammation in all the organs and tissues which constitute the uterine system, as elucidated by the application of physical investigation to the study of uterine diseases. The opportunities enjoyed by the author as house-physician to the Parisian hospitals St. Louis, Salpétriére, and Notre Dame de la Pitié, together with his connection with a large dispensary, have afforded him the means of accumulating experience that entitles him to be heard as an authority upon the subjects of which he treats. Dr. Bennett, however, is evidently a hobby rider; and although we doubt not he may have the discriminating tact and judgment that enable him to detect the existence of inflammation and to know when to apply his favorite remedy of cauterization aright, there is, to our minds, a danger that those into whose hands the work will fall, viz., the younger members of the profession, may not be equally fortunate, and hence ill effects may follow, as we know they have in one instance, from the incautious use of this agent. This, we conceive, to be the more likely to occur, from the circumstance that Dr. B. states in his preface, that he has endeavored to demonstrate "that inflammation is the keystone to uterine pathology, and that unless the phenomena which it occasions be recognised and taken into consideration, all is doubt, obscurity, and deception."

With this preliminary observation, by no means offered in disparagement of the able treatise before us, we proceed to lay before our readers an analysis of its contents.

The first two chapters are occupied with the consideration of some preliminary matters, and an excellent account of the anatomy and physiology of the uterus. Chapter III. is occupied in the consideration of acute metritis, in the non-puerperal state, and in the different form of acute, chronic and internal metritis. Chapter IV. treats of inflammation and abscess of the internal appendages in the puerperal and non-puerperal state. The next five chapters treat of inflammation and ulceration of the neck of the uterus in the virgin; during pregnancy; during and after abortion and parturition; in advanced life; and accompanying uterine polypi, and fibrous tumors of the uterus. Chapter XI. treats of inflammation of the vagina and vulva. Chapter XII. is on the connection between inflammation of the uterus, or its neck, and functional derangements and displacements of the uterus, leucorrhea, amenorrhea, dysmenorrhea, menorrhagia and uterine hemorrhage generally; sterility, abortion, prolapsus, anteversion, retroversion, retroflexion, chlorosis, hysteria.

In this chapter, a most valuable one by the way, a recapitulation of the facts presented to the reader in the preceding chapters is laid before him, thus presenting to him the real nature of those morbid states, in a large proportion of the cases in which they are observed.

Chapters XIII. and XIV. are devoted to the consideration of syphilitic ulceration of the neck of the uterus, and the diagnosis of cancer of this organ. Chapter XV. and last is occupied with the consideration of the treatment of the different forms of disease as enumerated in the preceding pages. In the treatment of simple inflammation of the neck of the uterus we are glad to have Dr. Bennett's testimony in favor of alum, which he declares to be the most efficacious of medicinal astringents next to nitrate of silver. It has always been a favorite with us, in the form of injection in the proportion of a drachm to a pint as recommended by Dr. B. The proper mode of administering vaginal injections is also insisted upon; viz., upon the back with the hips elevated. The mode in which such remedies are often employed either in the erect or sitting posture, is worse than useless.

We have said that the chief agent relied upon by Dr. Bennett in the treatment of these various inflammations was cauterization. It is not to be supposed, however, that this is his only treatment; the various adjuvants resorted to by every rational practitioner, as leeches, hip baths, injections, &c., are all employed by him. It would be in vain for us, in a limited article like the present, to do justice to the excellencies of Dr. Bennett's work; enough has been said and shewn of its contents to prove our favorable opinion of the doctrines and practice inculcated, with the cautions that we have thought it necessary to present to the young practitioner in relation to the use of caustics.

To the work is added an Appendix, "On the physical examination of the Uterus and its appendages," in which full and explicit directions are given for its conduction ; together with a synopsis of three hundred cases presenting uterine symptoms, treated at the Western Dispensary.

In relation to physical examination, either digital or specular, Dr. B. believes that the laudable sense of propriety which prevents our seeking such an exploration, is often carried much too far. In this we agree with him, taking it for granted that such an examination would not be hinted at where the attendant did did not conceive it to be absolutely necessary. It is this "laudable sense of propriety" that has been so sorely shocked by the recent efforts of a professor of obstetrics, in one of our schools, to teach demonstrative midwifery, and that has aroused such a storm of virtuous indignation against him. We confess we have been unable to discover wherein the enormity consists, and have not failed to uphold his course, believing, that conducted as we know his demonstrations were, such teaching cannot fail to prepare the student more completely for his responsible station, than any amount of didactic lectures. We commend to those who are disposed to view this matter in a different light, the manly and dignified defence of the proceeding as published under the editorial head of the March No. of the Buffalo Journal.

In concluding our notice of Dr. Bennett's work from which we have wandered a little, we desire to express our warm appreciation of its merits, and our sincere belief that it will not fail to be a most welcome guide to all who may be so fortunate as to secure it.

